# Where’s WALY? : A proof of concept study of the ‘wellbeing adjusted life year’ using secondary analysis of cross-sectional survey data

**DOI:** 10.1186/s12955-016-0532-5

**Published:** 2016-09-08

**Authors:** Rebecca Johnson, David Jenkinson, Chris Stinton, Sian Taylor-Phillips, Jason Madan, Sarah Stewart-Brown, Aileen Clarke

**Affiliations:** Division of Health SciencesWarwick Medical School, University of Warwick Coventry, Coventry, CV4 7AL UK

**Keywords:** EQ-5D, WALY, Wellbeing, WEMWBS

## Abstract

**Background:**

The Quality-Adjusted Life Year (QALY) is a measure that combines life extension and health improvement in a single score, reflecting preferences around different types of health gain. It can therefore be used to inform decision-making around allocation of health care resources to mutually exclusive options that would produce qualitatively different health benefits. A number of quality-of-life instruments can be used to calculate QALYs. The EQ-5D is one of the most commonly used, and is the preferred option for submissions to NICE (https://www.nice.org.uk/process/pmg9/). However, it has limitations that might make it unsuitable for use in areas such as public and mental health where interventions may aim to improve well-being. One alternative to the QALY is a Wellbeing-Adjusted Life Year. In this study we explore the need for a Wellbeing-Adjusted Life Year measure by examining the extent to which a measure of wellbeing (the Warwick-Edinburgh Mental Well-being Scale) maps onto the EQ-5D-3L.

**Methods:**

Secondary analyses were conducted on data from the Coventry Household Survey in which 7469 participants completed the EQ-5D-3L, Warwick-Edinburgh Mental Well-being Scale, and a measure of self-rated health. Data were analysed using descriptive statistics, Pearson’s and Spearman’s correlations, linear regression, and receiver operating characteristic curves.

**Results:**

Approximately 75 % of participants scored the maximum on the EQ-5D-3L. Those with maximum EQ-5D-3L scores reported a wide range of levels of mental wellbeing. Both the Warwick-Edinburgh Mental Well-being Scale and the EQ-5D-3L were able to detect differences between those with higher and lower levels of self-reported health. Linear regression indicated that scores on the Warwick-Edinburgh Mental Well-being Scale and the EQ-5D-3L were weakly, positively correlated (with R^2^ being 0.104 for the index and 0.141 for the visual analogue scale).

**Conclusion:**

The Warwick-Edinburgh Mental Well-being Scale maps onto the EQ-5D-3L to only a limited extent. Levels of mental wellbeing varied greatly amongst participants who had the maximum score on the EQ-5D-3L. To evaluate the relative effectiveness of interventions that impact on mental wellbeing, a new measure – a Wellbeing Adjusted Life Year – is needed.

## Background

When making decisions about interventions it is important to consider their effect on both length of life and the quality of that life. One way in which this is achieved is through the use of Quality-Adjusted Life Years (QALY). The most common method of calculating QALYs uses a measure called EQ-5D-3L [1, 2] which has been successfully employed to assess the relative effectiveness of a wide range of treatments and interventions. However, there is evidence of ceiling effects in the EQ-5D-3L, with up to 85 % of respondents who have physical health problems reporting maximum scores [3, 4]. Further, there are questions about whether the EQ-5D-3L is appropriate for assessing the impacts of conditions such as hearing loss, age-related macular degeneration, diabetic retinopathy and psychotic disorders. This is because of problems such as failure to detect differences in quality of life between people with different stages of disease severity, and a limited ability to detect improvements in quality of life following interventions [4–9].

Wellbeing is now recognised as a determinant of longevity and an important player in the adoption and maintenance of healthy lifestyles and successful management of chronic illness [10]. The case for improving wellbeing has been made on both health and economic grounds [11]. Interventions to promote mental wellbeing (e.g. parks and gardens, crime reduction, art festivals, cookery clubs, wellbeing festivals, Tai Chi, yoga, sports) may be offered in many different sectors, both public and private and it is important to be able to assess their relative effectiveness compared to interventions offered in the health sector. While researchers have mapped utility of the EQ-5D-3L onto utilities derived from a range of health outcome measures (e.g. SF-6D) [12–14], there has been little research on how to address the cost-utility of interventions aimed at improving mental wellbeing. If wellbeing is a concept that substantially extends existing concepts of health, then a health-related measure of quality-of-life will underestimate the benefit of interventions that improve wellbeing. In a time of austerity, this is clearly an issue for public health commissioning. One approach to address the cost-utility of these types of interventions could be to develop a wellbeing adjusted life year (WALY).

A well-established tool to measure mental wellbeing is the Warwick-Edinburgh Mental Well-being Scale (WEMWBS) [15, 16]. Full details of the WEMWBS are available (www2.warwick.ac.uk/fac/med/research/platform/wemwbs/). In brief, WEMWBS was developed to meet the need for a robust, population-based measure of mental wellbeing to evaluate programmes and monitor mental wellbeing at the population level [15]. WEMWBS has been in use since 2007, it is valid and reliable at the population level [15] and is sensitive to change [17]. Originally validated in English and Scottish populations of people aged 16 and older [15], the scale has now been translated into many different languages and validated in many different cultures [18]. It has been successfully used to measure wellbeing outcomes in a range of health interventions [19–25]. Evidence suggests that users of mental health services and their carers prefer the WEMWBS to other health outcome measures [26].

WEMWBS is gaining momentum as a useful tool in public health practice, particularly since its inclusion as a measure in the Scottish Governments Outcomes Framework [27] and the English Public Health Outcomes Framework [28]. However, there is no underlying research on how to assess the cost-effectiveness of interventions using this tool. In this study we explore the extent to which the WEMWBS and the EQ-5D-3L estimate the health state value of individuals with different levels of mental wellbeing (i.e. whether WEMWBS “maps” onto the EQ-5D-3L) [29]. If mapping is poor, this suggests that there is a need to develop a Wellbeing Adjusted Life Year (WALY).

## Methods

### Setting

Data used in this study come from a survey of residents of Coventry, UK. The health of people who live in Coventry is worse than that for England overall [30]. The gap in life expectancy between men and women is the widest in the West Midlands (approximately 9 years difference between the least deprived and most deprived areas of Coventry). Compared to the average for England, there are more early deaths from cancer, more hospital stays for self-harm, a significantly greater proportion of obese adults and obese children, and significantly lower rates of physical activity among adults in Coventry. Smoking during pregnancy, and alcohol-related hospital admissions are both higher than the average in England [30]. The Coventry Household Survey (CHS) has measured environment, lifestyle behaviours, and mental wellbeing and health related quality of life (using the EQ-5D-3L) since 2011 [31–33].

### Design and participants

Secondary analysis of cross-sectional survey data taken from the CHS at three time points (2011, 2012, and 2013) [31–33] was conducted. Participants were residents of Coventry who were aged 16 years or older at the time of the survey. There were 3144 participants in the 2011 survey, 2117 participants in the 2012 survey, and 2208 participants in the 2013 survey. No person was surveyed in more than 1 year so data were combined for analyses (*n* = 7469).

### Data collection

Data were obtained from the CHS which comprises 45 questions in six topic sections: community and neighbourhood, environment and housing, crime and safety, work and training, transport and accessibility, and health and wellbeing. Households were sampled using a stratified sampling approach [34]. The Royal Mail Postcode Address File was used to obtain a full list of addresses in Coventry, which was linked to the Middle Super Output Areas (MSOA). Three postcodes (one random and two numerically next-nearest) were sampled within each of the 42 MSOA to ensure representativeness based on deprivation levels, consistent with the overall population of Coventry. This resulted in 126 primary sampling points. Interviewers used age sampling, asking to speak to the ‘household member whose birthday is next’. Approximately 200 additional surveys were conducted around Coventry city centre in order to represent mobile populations. Survey questions were asked by face-to-face interview, with responses recorded by the interviewer, except for WEMWBS which was self-completed. The survey took approximately 20 min to complete. Data collection was undertaken by the research consultancy firms BMG and MEL using teams of trained, multi-language interviewers. A 10 % sample of each interviewer’s survey batch was checked. A further 10 % of survey participants were contacted to ensure that interviews had taken place as recorded. Data were then anonymised. Data entry, primary coding and cleaning/consistency checks were undertaken.

### Measures

#### EQ-5D-3L

The EQ-5D-3L is a generic preference-based measure used to assess health-related quality of life and cost effectiveness of health interventions [1]. It measures five dimensions: mobility, self-care, usual activities, pain/discomfort, and anxiety/depression. Each dimension has three levels which are scored as a ‘1’ (e.g. ‘I have no problems in walking about’), ‘2’ (e.g. ‘I have some problems in walking about’), or ‘3’ (e.g. ‘I am confined to bed’). Each dimension is coded and together comprises an end health state such as ‘11111’ (in this example, no problems in any of the health dimensions are indicated). There are 243 possible health states that are relevant for both clinical and general populations. The EQ-5D-3L utility index uses a time trade-off method For the UK, this is from a sample of 3395 respondents from the general population [12, 13] Intra-class coefficients (ICC) of 0.78 have been reported for the Visual Analogue Scale (VAS) and 0.73 for Time Trade Off (TTO) methods, with little non-response effect [12, 35]. The EQ-5D-3L has been used extensively and translated into at least 171 languages (http://www.euroqol.org/). It has been found to be a practical way of measuring and detecting differences in the health states of individuals within general [36, 37] and patient populations [38, 39].

#### WEMWBS

WEMWBS is a self-reported measure of mental wellbeing [15]. It is a positively worded 14-item scale covering hedonic (eg I’ve been feeling cheerful’) and eudaimonic (eg ‘I have been feeling useful’) components of mental wellbeing. For each item, participants can select a response option from ‘none of the time’ (item score = 1) to ‘all of the time’ (item score = 5) with a 2 week timeframe. The scale is scored by adding up each item for a total score ranging from 14 to 70. It has been found valid and reliable (www2.warwick.ac.uk/fac/med/research/platform/wemwbs/) [15]. WEMWBS was validated using eight scales that incorporated similar concepts or were likely to be associated with mental wellbeing [15]. Data on mental ill health were collected, and social desirability bias was assessed using the Balanced Inventory of Desirable Responding (BIDR) [40]. Content validity was assessed and all response categories were used at least once by respondents, with little evidence of skew within the distributions of each item response. Construct validity was assessed using confirmatory factor analysis with least squares estimation. Both the goodness of fit index (0.91) and adjusted goodness of fit index (0.87) were satisfactory, and the Root Mean Square Error of Approximation was within the desired upper limit (0.0502). Good internal consistency was demonstrated (Cronbach’s alpha 0.89 and 0.91 in each sample). There was no evidence of floor or ceiling effects. There were low to moderate correlations with overall health, as measured by EQ-5D VAS (*r* = 0.43, *p* < 0.01) and high correlations with scales measuring aspects of wellbeing or positive affect, such as the Positive and Negative Affect Schedule-Positive Affect Scale, [41] among others (Positive Affect *r* = 0.71, *p* < 0.01). Equally, there was a negative correlation between Positive and Negative Affect Schedule-Negative Affect Scale and WEMWBS (*r* = −0.54, *p* < 0.01) [15].

#### Self-rated health

Self-rated health (SRH) is a generic health measure used in a range of populations and countries [42–45], and has been has been associated with all-cause mortality [44, 46]. Self-rated health was measured by asking participants ‘How would you say your health is, in general?”. Response options ranged from ‘very good’ to ‘very bad’. SRH has demonstrated moderate test-retest reliability [43], and has consistent and strong predictive validity with respect to mortality [47, 48].

### Statistical analysis

Analyses were conducted using R, version 3.0.3 [49], with the pROC package [50]. To address health inequalities, analysis was stratified by age, gender, and socioeconomic status. Descriptive statistics and plots were used to explore the distributions of, and the relationships between, WEMWBS and the EQ-5D-3L on each dimension, the visual analogue scale (VAS), and the preference-based index. We assessed the correlation between the EQ-5D-3L VAS and WEMWBS. Floor and ceiling effects were estimated by calculating the proportion of responses at the lowest and highest possible level for each dimension of both the EQ-5D-3L and WEMWBS.

We calculated the area under curve (AUC) of the receiver operating characteristic (ROC) curve to examine whether WEMWBS and the EQ-5D-3L were able to distinguish between participants with ‘very good’ vs lower self-rated health.

WEMWBS was mapped onto the EQ-5D-3L using methodology derived from Longworth and Rowen [29]. Since we were only interested in whether WEMWBS maps onto the EQ-5D-3L no other variables were included in the model. The model fit for linear models was assessed using the R^2^ statistic.

## Results

### Sample characteristics

The demographics of the participants, in terms of age, sex and index of multiple deprivation (IMD) quintiles are shown in Table 1.

**Table 1:** Demographics of participants

		2011	2012	2013	Total	Percent
Age (years)	16–24	521	358	342	1221	16.3 %
	25–34	533	378	428	1339	17.9 %
	35–44	462	378	320	1160	15.5 %
	45–54	394	293	310	997	13.3 %
	55–64	346	303	322	971	13.0 %
	65–74	178	266	289	733	9.8 %
	75 and over	195	138	178	511	6.8 %
	Not available	515	3	19	537	7.2 %
Gender	Male	1547	1020	1061	3628	48.6 %
	Female	1597	1095	1147	3839	51.4 %
	Not available	0	2	0	2	0.0 %
IMD Quintile	1st Quintile	1179	690	420	2289	30.6 %
	2nd Quintile	798	607	417	1822	24.4 %
	3rd Quintile	559	335	421	1315	17.6 %
	4th Quintile	392	337	505	1234	16.5 %
	5th Quintile	216	148	445	809	10.8 %

### Descriptive statistics

**Fig. 1:**
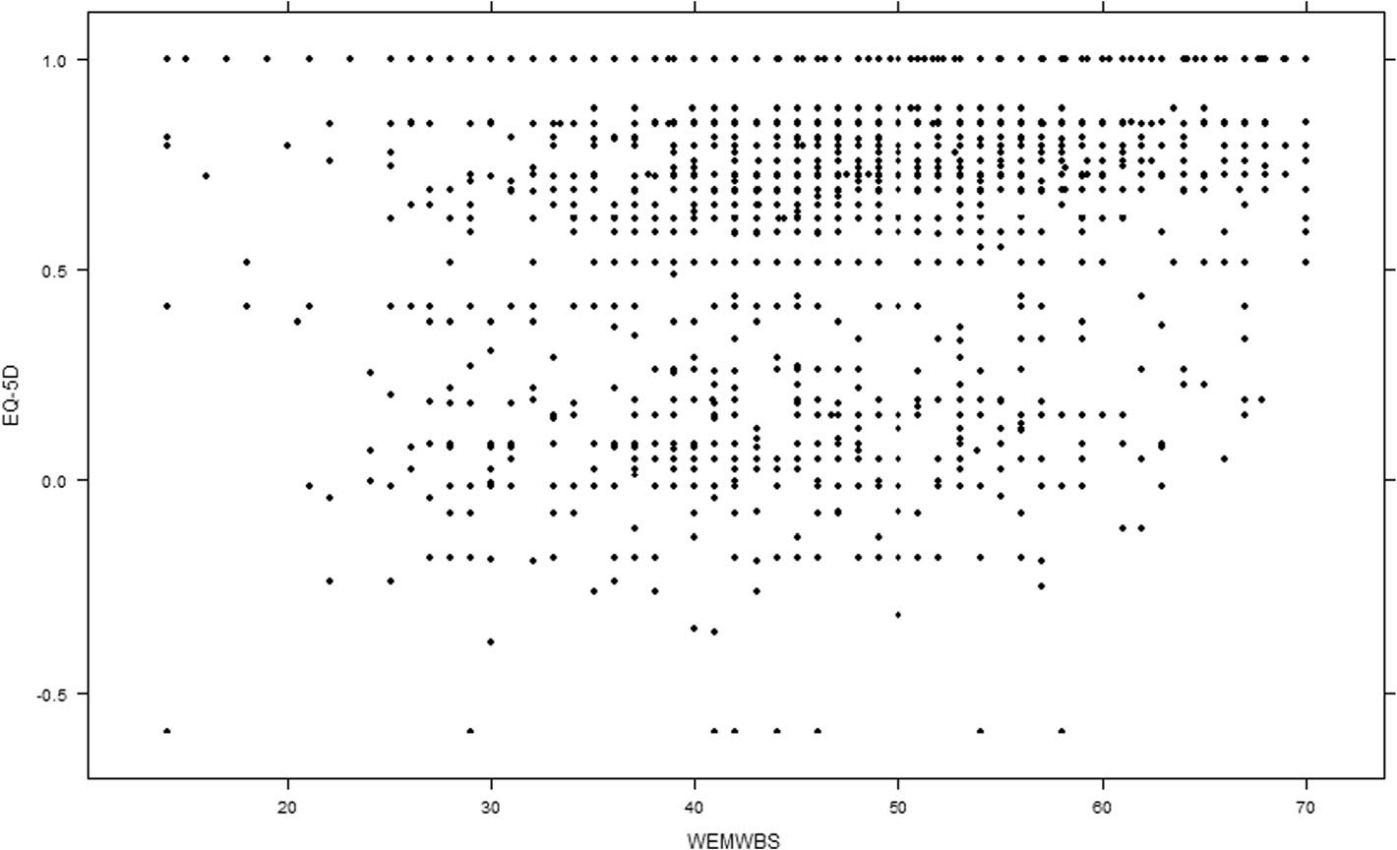
Scatter plot of WEMWBS and EQ-5D-3L scores

The joint distribution of the EQ-5D-3L and WEMWBS scores is shown in Table 2 and Fig. 1. Marginal distributions show that 74.3 % of the participants had an EQ-5D-3L score of 1, i.e. three out of four participants’ EQ-5D-3L scores were clustered at the top most level of the scale where no further measurement would be recorded. This demonstrates a large ceiling effect for the EQ-5D-3L. The marginal distribution of the WEMWBS scores was closer to symmetric, showing a more normal distribution and a wider range of possible WEMWBS scores. The mean EQ-5D-3L score was 0.90, with a standard deviation of 0.23. The mean WEMWBS score was 52.36, with a standard deviation of 8.85. Correlations between scores on the two measures were *r* = 0.322 (95 % CI: 0.301, 0.342) and r_s_ = 0.299 (95 % CI: 0.275, 0.320). Correlations between WEMWBS and EQ-5D-3L, stratified by age, gender, socioeconomic status, are shown in Table 3.

**Table 2:** Joint distribution of WEMWBS and EQ-5D-3L, and WEMWBS and EQ-5D-3L Visual Analogue Scale

	WEMWBS										
EQ-5D-3L	(14,21]	(21,28]	(28,35]	(35,42]	(42,49]	(49,56]	(56,63]	(63,70]	NA	Total	%
(-0.6,0]	1	13	17	29	29	16	9	0	4	118	1.6 %
(0,0.2]	0	9	21	48	43	38	17	4	3	183	2.5 %
(0.2,0.4]	1	5	6	15	14	9	5	4	1	60	0.8 %
(0.4,0.6]	3	5	11	54	60	41	11	9	4	198	2.7 %
(0.6,0.8]	2	11	36	117	268	303	136	39	11	923	12.4 %
(0.8,1)	0	7	19	71	115	108	49	23	7	399	5.3 %
1	5	13	41	385	1032	2119	1193	677	87	5552	74.3 %
NA	1	1	1	7	4	9	3	1	9	36	0.5 %
Total	13	64	152	726	1565	2643	1423	757	126	7469	
%	0.2 %	0.9 %	2.0 %	9.7 %	21.0 %	35.4 %	19.1 %	10.1 %	1.7 %		
VAS											
(0,50]	5	38	76	189	236	187	66	25	19	841	11.3 %
(50,60]	0	7	14	68	143	120	50	18	4	424	5.7 %
(60,70]	3	2	12	118	217	279	125	48	9	813	10.9 %
(70,75]	0	3	5	41	133	156	80	30	4	452	6.1 %
(75,80]	2	7	20	102	273	524	268	117	23	1336	17.9 %
(80,85]	0	2	2	33	84	170	105	49	6	451	6.0 %
(85,90]	1	1	5	54	223	523	331	165	18	1321	17.7 %
(90,95]	0	0	3	17	65	202	115	71	7	480	6.4 %
(95,100]	2	1	6	30	87	252	195	150	9	732	9.8 %
NA	0	3	9	74	104	230	88	84	27	619	8.3 %

**Table 3:** Correlation’s between EQ-5D-3L and WEMWBS within each level of the variables age, gender and IMD

		Pearson		Spearman	
			95 % Confidence		95 % Confidence
		Estimate	Interval	Estimate	Interval
Age	16–24	0.238	(0.184,0.290)	0.224	(0.169,0.278)
	25–34	0.300	(0.251,0.349)	0.252	(0.200,0.303)
	35–44	0.312	(0.259,0.364)	0.318	(0.264,0.371)
	45–54	0.344	(0.288,0.398)	0.269	(0.209,0.327)
	55–64	0.389	(0.333,0.441)	0.370	(0.312,0.425)
	65–74	0.313	(0.246,0.377)	0.274	(0.204,0.341)
	75 and over	0.289	(0.206,0.367)	0.350	(0.268,0.427)
Gender	Male	0.302	(0.272,0.332)	0.275	(0.244,0.306)
	Female	0.334	(0.305,0.362)	0.316	(0.286,0.345)
IMD	1st Quintile	0.308	(0.270,0.345)	0.277	(0.238,0.316)
	2nd Quintile	0.319	(0.277,0.360)	0.296	(0.253,0.339)
	3rd Quintile	0.324	(0.274,0.372)	0.292	(0.240,0.342)
	4th Quintile	0.344	(0.293,0.392)	0.298	(0.245,0.349)
	5th Quintile	0.336	(0.272,0.396)	0.362	(0.298,0.422)

**Fig. 2:**
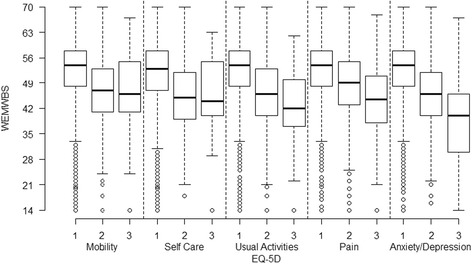
Distribution of the WEMWBS scores for each level of each domain of EQ-5D-3L

The joint distribution of WEMWBS with the EQ-5D-3L VAS score is shown in Table 2. The mean EQ-5D-3L VAS score was 77.5, with a standard deviation of 18.4. The median score was 80, with quartiles of 70 and 90. Correlations between the EQ-5D-3L VAS score and WEMWBS were *r* = 0.375 (95 % CI: 0.355, 0.396) and r_s_ = 0.355 (95 % CI: 0.333, 0.376). The distributions of the WEMWBS scores for each level of each domain of EQ-5D-3L are illustrated in Fig. 2, showing there is a wide spread of WEMWBS scores within each EQ-5D-3L domain.

### Construct validity of EQ-5D-3L and WEMWBS

#### Self-rated health

**Fig. 3:**
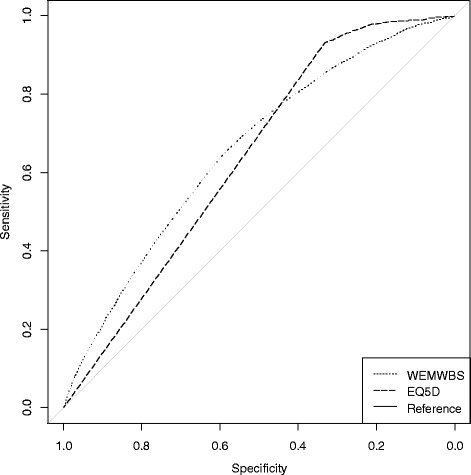
Roc curves for WEMWBS and EQ-5D-3L predicting very good self-report health

Nearly one third (30.5 %) of participants reported ‘very good’ self-rated health. The WEMWBS and EQ-5D-3L were both able to distinguish between participants with ‘very good’ and less than very good self-rated health: WEMWBS (AUC 0.657 [0.643, 0.670]) and EQ-5D-3L (AUC 0.636 [0.628, 0.644]). If a participant with very good and a participant with less than very good self-rated health were randomly chosen from the population, WEMWBS has a slightly higher probability than EQ-5D-3L of ranking a participant with very good self-rated health higher than one with less than very good self-rated health due to the ceiling effects of EQ-5D-3L as shown by the higher ROC curve to the left of the plot (Fig. 3).

### Mapping WEMWBS onto the EQ-5D-3L

WEMWBS predicted EQ-5D-3L and EQ-5D-3L VAS scores to a limited extent, with adjusted R^2^ statistics of 0.104 and 0.141, respectively (Table 4).

**Table 4:** Linear models of WEMWBS and EQ-5D-3L, and WEMWBS and EQ-5D-3L Visual Analogue Scale

Model		Estimate	95 % CI		*p*-value	
EQ-5D-3L^a^	Constant	0.468	0.438	0.498	<0.001	
	WEMWBS	0.0082	0.0077	0.0088	<0.001	
EQ-5D-3L VAS^b^	Constant	36.4	33.9	38.8	<0.001	
	WEMWBS	0.786	0.740	0.832	<0.001	
Model	ANOVA	Sum of Squares	Degrees of Freedom	Mean Square	F statistic	*p*-value
EQ-5D-3L^a^	Regression	38.86	1	38.86	846.84	<0.001
	Residuals	336.33	7329	0.046		
	Total	375.19	7330		R^2^	0.104
EQ-5D-3L VAS^b^	Regression	1	324278	324278	1110.8	<0.001
	Residuals	6768	1975775	292		
	Total	6769	2300053		R^2^	0.141

^a^7331 valid cases

^b^6770 valid cases

Linear models indicated that WEMWBS scores explained 10.4 % of the variability in the EQ-5D-3L scores and 14.1 % of the variability in the EQ-5D-3L VAS scores.

## Discussion

The aim of this study was to assess the extent to which WEMWBS maps onto the EQ-5D-3L. Consistent with previous studies [14], we found a pronounced ceiling effect in the EQ-5D-3L, with nearly three quarters of participants having the maximum score of 1 (i.e. the best possible health-related quality of life). No ceiling effect was observed for WEMWBS. WEMWBS scores spanned the whole range of possible values (14–70), with a mean of 53.9 for participants who had a score of 1 on the EQ-5D-3L. This suggests that WEMWBS and the EQ-5D-3L are not measuring the same construct and that there is scope for improving mental wellbeing of individuals who have maximum scores on this quality of life measure. WEMWBS was positively correlated with both the EQ-5D-3L and the EQ-5D-3L VAS for the sample as a whole and when stratified by age, sex, and socioeconomic status, though this correlation was quite low. Both WEMWBS and the EQ-5D-3L detected differences between those with very good versus other levels of self-reported health, but neither measure was especially good at detecting these differences. This is not surprising as the constructs that they are measuring are not identical. WEMWBS explained a very limited amount of the variability of the EQ-5D-3L and the EQ-5D-3L could not assess with any precision the effectiveness of interventions to promote mental wellbeing relative to other health related interventions.

It is essential that the preference-based measure adopted by an economic evaluation captures all consequences of the alternatives being evaluated that might materially affect the net benefit of each alternative to the decision-maker. It can be argued that wellbeing is a concept that extends existing concepts of health, prompting the need for a health-related measure of quality-of-life that will not underestimate the benefit of interventions that improve wellbeing. This remains an issue for interventions in sectors such as social care and education, and is increasingly relevant for public health and mental health interventions [27, 28]. The EQ-5D-3L has been shown to capture the impact of health care interventions for a broad range of conditions, but the fact that we found a ceiling effect in the EQ-5D-3L (as have others before us [51], with nearly three quarters of participants at the maximum score reinforces the likelihood that it does not capture relevant changes that matter to individuals or, therefore, to economic evaluations [51]. The EQ-5D-3L is preference-based, i.e. tariffs exist that reflect societal preferences for different types of health gain, relative to life extension, to permit calculation of QALYs. If a similar tariff existed for WEMWBS that allowed estimation of Wellbeing Adjusted Life Year (WALYs) gained, this could be used to support priority-setting within and across sectors in a way that reflects societal preferences more appropriately. Further research would be required to understand how the overlap between health and wellbeing varies in different populations, and to determine how the QALY and/or WALY can be used to value the benefits of interventions in these populations, while avoiding double-counting.

A limitation of our study is that we compared WEMWBS to the EQ-5D-3L. A new version (EQ-5D-5L) has recently been published [52] which has ameliorated some of the limitations of EQ-5D-3L discussed in this paper and has reduced ceiling effects with increased discriminatory power [53].

WEMWBS has the potential to be used as the basis of a preference-based measure to evaluate and prioritise public sector interventions between and within sectors, including traditional health related interventions. However, it has not yet been used to inform priority-setting and a preference-based tariff does not currently exist. The next step for our research is to develop and evaluate the utility of a well-being adjusted life year (WALY) based on WEMWBS. The stages in the development of the WALY will include a valuation exercise to generate a preference tariff for WEMWBS, the identification of an appropriate preference elicitation technique for wellbeing states, and exploration of the variation in valuations across samples.

## Conclusions

There is wide variation in the levels of mental wellbeing amongst participants with very high levels of self-reported health who score at ceiling level on the EQ-5D-3L with evidence of limited mapping of WEMWBS onto EQ-5D-3L. These results suggest that the two measures examine related, but not identical, aspects of quality of life. We propose exploration of the feasibility, appropriateness, and practicality of a Wellbeing-Adjusted Life Year.

## Abbreviations

CHS: Coventry household survey
MSOA: Middle super output areas
QALY: Quality adjusted life year
WALY: Wellbeing adjusted life year
WEMWBS: Warwick-Edinburgh mental well-being scale
